# A comparison of anterior reconstruction of spinal defect using nano-hydroxyapatite/polyamide 66 cage and autologous iliac bone for thoracolumbar tuberculosis: a stepwise propensity score matching analysis

**DOI:** 10.3389/fbioe.2024.1376596

**Published:** 2024-05-10

**Authors:** Qiujiang Li, Peng Xiu, Xi Yang, Lei Wang, Limin Liu, Yueming Song

**Affiliations:** Department of Orthopedic Surgery, West China Hospital, Sichuan University, Chengdu, China

**Keywords:** spinal tuberculosis, nano-hydroxyapatite/polyamide 66, iliac bone, N-HA/PA66, propensity score matching

## Abstract

**Purpose:**

Previous studies have confirmed the advantages and disadvantages of autogenous iliac bone and nano-hydroxyapatite/polyamide 66 (n-HA/PA66) cage. However, there is no conclusive comparison between the efficacy of the two implant materials in spinal tuberculosis bone graft fusion. The aim of this study was to analyze the mid-to long-term clinical and radiologic outcomes between n-HA/PA66 cage and autogenous iliac bone for anterior reconstruction application of spinal defect for thoracolumbar tuberculosis.

**Methods:**

We retrospectively reviewed all patients who underwent anterior debridement and strut graft with n-HA/PA66 cage or iliac bone combined with anterior instrumentations between June 2009 and June 2014. One-to-one nearest-neighbor propensity score matching (PSM) was used to match patients who underwent n-HA/PA66 cage to those who underwent iliac bone. Clinical outcomes were assessed using the Japanese Orthopaedic Association (JOA) and visual analogue score (VAS). Radiographic evaluations included cage subsidence and segmental angle.

**Results:**

At the end of the PSM analysis, 16 patients from n-HA/PA66 cage group were matched to 16 patients in Iliac bone group. The C-reactive protein (CRP) and erythrocyte sedimentation rate (ESR) values in the n-HA/PA66 group decreased significantly from 33.19 ± 10.89 and 46.63 ± 15.65 preoperatively, to 6.56 ± 2.48 and 9.31 ± 3.34 at the final follow-up, respectively (*p* < 0.001). There were no significant differences in the CRP and ESR values between the two groups at the final follow-up. The VAS and JOA scores in the iliac bone and n-HA/PA66 group were significantly improved at the 3-month follow-up postoperatively (both *p* < 0.001). Then, improvements of VAS and JOA scores continue long at final follow-up. However, there were no significant differences in the VAS and JOA scores at any time point between the two groups (*p* > 0.05). Although the segmental angle (SA) significantly increased after surgery in both groups, there was no significant difference at any time point after surgery (*p* > 0.05). There were no significant differences in the cage subsidence and fusion time between the two groups.

**Conclusion:**

Overall, our data suggest that the n-HA/PA66 cage outcomes are comparable to those in the autogenous iliac bone, with a similar high fusion rate as autogenous iliac bone.

## Introduction

With advances in population aging, the incidence of spinal tuberculosis in elderly people has been rising year by year (Held et al., 2018; Ramakrishnan et al., 2022). Spinal tuberculosis is an inflammatory disease caused by *Mycobacterium tuberculosis*, the most common symptom of which is low back pain, with corresponding radiating pain in the lower limbs and neurological dysfunction (Dunn and Ben, 2018; Khanna and Sabharwal, 2019; Chipeio et al., 2021). If not treated in time, it will cause spinal deformity, even lead to severe consequences such as lower limb paralysis, seriously affecting the quality of life of patients. Anterior debridement, bone graft fusion and internal fixation is still one of the standard surgical procedures for spinal tuberculosis because of its direct and thorough debridement and convenient and reliable spinal reconstruction (Yilmaz et al., 1999; Lu et al., 2012; Wang et al., 2017). The key to successful operation is to repair the defect of vertebral body and reconstruct the stability of spinal column. The traditional method is mainly to use autogenous iliac bone or artificial materials for bone grafting to achieve spinal stabilization and promote bone healing. However, there are some problems with these traditional methods. Autogenous iliac bone transplantation may lead to increased operative time and risk, postoperative complications such as infection and pain, and bone healing problems, especially in cases where large bone grafts are required, and the available bone material is limited, which may affect rehabilitation outcomes. Artificial implants may suffer from insufficient biocompatibility and osseointegration, affecting their stability and long-term results, and may carry a higher risk of infection and complications requiring reoperation, such as loosening or fracture (Gao et al., 2017; Suya et al., 2019; Wu et al., 2021). In recent years, a new type of bone graft material-nano-hydroxyapatite/polyamide 66 (n-HA/PA66) bioactive cage has been widely used in bone graft fusion surgery, its advantages are good biocompatibility, bioactivity, can promote bone healing, but also can avoid the use of traditional methods such as autoiliac bone may exist problems (Hu et al., 2019; Deng et al., 2022; Li et al., 2023; Zhang et al., 2023). Although the advantages and disadvantages of autogenous iliac bone and n-HA/PA66 cage have been extensively studied, there is no conclusive comparison between the efficacy of the two implant materials in spinal tuberculosis bone graft fusion. Thus, in this study, a confounder-elimination process was conducted using propensity score matching (PSM) to compare the clinical outcomes between n-HA/PA66 cage and autogenous iliac bone for anterior reconstruction application of spinal defect for thoracolumbar tuberculosis.

## Material and methods

### Study design

This was a retrospective cohort study that was conducted at a single center. The study included all patients who underwent anterior debridement and strut graft with n-HA/PA66 or iliac combined with anterior instrumentations between June 2009 and June 2014. Thereafter, patients were categorized into 2 groups based on the strut graft used, and stepwise PSM was implemented to guarantee matching baseline data between groups. This study was approved by our institutional review board and local ethics committee (No. 2019-654). Written informed consent was obtained from all participants.

### Patient selection

This retrospective study involved all patients who underwent anterior debridement and strut graft with n-HA/PA66 cage or iliac bone combined with anterior instrumentations between June 2009 and June 2014. Inclusion criteria were as follows: ①Patients were confirmed with diagnosis of thoracolumbar spinal tuberculosis based on a comprehensive physical examination, laboratory examination, and imaging examination including CT and MRI; ②Tuberculosis was confirmed by postoperative lesions histopathological examination or pus bacterial culture; ③Serious or progressive neurological impairment; ④Intractable or worsening pain, and clinical symptoms were not obviously relieved after standard anti-tuberculosis treatment; ⑤Spinal instability or serious, progressive kyphotic deformity; ⑥No history of spine surgery. Exclusion criteria were as follows:①Patients with obvious contraindications to surgery; ②Patients with active, not effectively controlled, and strong infectivity pulmonary tuberculosis; ③Patients with unclear efficacy of anti-tuberculosis treatment or resistance to anti-tuberculosis medication. ④Patients lacking complete clinical follow up data or follow up duration less than 3 years after surgery. Finally, there were 63 patients with complete clinical data enrolled.

### Preoperative management

All patients received anti-tuberculosis chemotherapy that included rifampicin, isoniazid, pyrazinamide and ethambutol for at least 6 weeks before surgery. All patients were asked to rest, local immobilization, and strengthen nutritional support. At the same time, liver protection drugs were taken orally, and liver and renal functions and inflammatory indexes such as blood routine, C-reactive protein (CRP) and erythrocyte sedimentation rate (ESR) were reexamined regularly. Most of the patients were operated after CRP<40 mg/L. For the patients with obvious paralysis progression, ESR and CPR were not absolutely required to be controlled to a certain level. The improvement of general condition was the main reference to determine the optimal timing of operation.

### Surgical procedures

All patients included in the study underwent surgery by the same spinal surgery team. Lateral position was taken after general anesthesia. An anterior approach was utilized for accessing the thoracic spine (T3-T10), while the thoracolumbar segments would require the removal of the 11th or 12th rib via the thoracic or extrapleural retroperitoneal approach. For lumbar tuberculosis, an extraperitoneal approach was employed. Complete removal of granulation tissue, caseous material, necrotic intervertebral disc tissue, and dead bone is necessary to relieve compression on the dural sac. After thorough irrigation with saline solution, appropriate length and size of n-HA/PA66 or autologous iliac bone would be inserted into the intervertebral space. Hollow n-HA/PA66 would be filled with autogenous bone graft. The selection of internal fixation is based on the intraoperative fixation of the vertebrae and segments. Typically, a single screw-rod construct is used for the mid-upper thoracic vertebrae, while a double screw-rod construct is preferred for the thoracolumbar segments to achieve better rotational stability. Thoracic closed drainage tube or conventional drainage tube was placed after repeated flushing, and the incision was closed layer by layer.

### Postoperative management

After the drainage tube was pulled out, functional exercise was performed under the protection of thoracolumbar brace. The patients were treated with isoniazid, rifampicin, pyrazinamide and ethambutol for at least 12 months. Blood biochemical indexes and liver and kidney functions were reexamined regularly, and postoperative complications such as anemia and hypoproteinemia were corrected in time.

### Clinical assessment

All patient-related information is obtained from medical records. Low back pain and neurological status were assessed using the visual analogue scale (VAS) and Japanese Orthopaedic Association (JOA) preoperatively, at 3-month postoperatively, final follow-up. Neurological functions were evaluated using the ASIA classification. All patients follow-up information was collected during outpatient visits or telephone follow-up.

### Radiographic measurements

Radiological examinations were performed at preoperative, postoperative 1 week and at least 3 years postoperative follow-up. Static and lateral flexion/extension X-ray were conducted to assess the radiological parameters (cage subsidence and segmental angle). Segmental angle: the angle formed between the superior endplate of the upper vertebral body and the inferior endplate of the lower vertebral body. Postoperative 3-dimensional (3D) CT scans were used to assess bony fusion that the formation of trabeculation between the bone autograft inside the strut and the adjacent endplates, following the method of Shah et al. (Shah et al., 2003). The patients were followed up every 3 months for the first years and then yearly thereafter.

### Propensity score matching

To eliminate selection bias, PSM analysis was utilized to select and match patients from the n-HA/PA66 group to patients in the iliac bone group. Variables that were deemed to have an effect on postoperative fusion rate and cage subsidence were chosen for the PSM multivariable logistic regression model. These variables included age, sex, number of segments, and follow-up time. The matching technique used was one-to-one nearest neighbor matching with a match tolerance of 0.2 without replacement, employing the “MatchIt” package in R as previously described by Austin (Austin, 2011).

### Statistical analysis

Continuous variables were presented as mean ± standard deviation (SD) for normally distributed data, while for non-normally distributed data, they were expressed as median (M) and interquartile range (IQR). If two groups of data were normally distributed, Student’s t-test was used to compare the differences, otherwise Wilcoxon rank-sum test was used. Categorical data were expressed as percentages, and the Chi-square test or Fisher exact test was used to analyze the differences between groups. The statistical analyses were performed using SPSS 26.0 (SPSS Inc., Chicago, IL) and R version 4.0.4 (R Project for Statistical Computing). A *p*-value less than 0.05 was considered statistically significant.

## Results

At the end of the PSM analysis, 16 patients from n-HA/PA66 cage group were matched to 16 patients in Iliac bone group. All patients successfully completed one-stage lesion debridement, n-HA/PA66 cage or Iliac bone placement and fixation. After PSM, all demographic data and clinical characteristics were similar between groups (*p* > 0.05) (Table 1). The mean age for the whole cohort was 42.66 ± 11.99 years old, 16 (50.00%) were female and 16 (50.00%) were male. The average follow-up time for 32 patients was 45.03 ± 8.00 months. The mean operating time was 192.97 ± 48.77 min, intraoperative blood loss was 427.19 ± 112.31 mL. The detailed demographic data after PSM are displayed in Table 1.

**TABLE 1:** Comparison of preoperative demographics between the two group.

	Whole group (n = 32)	Iliac bone group (n = 16)	n-HA/PA66 group (n = 16)	t/χ^2^	*p*-value
Age, years	42.66 ± 11.99	43.50 ± 12.64	41.81 ± 11.65	−0.393	0.697
Gender, %				0.500	0.480
Male	16 (50.00%)	9 (56.25%)	7 (43.75%)		
Female	16 (50.00%)	7 (43.75%)	9 (56.25%)		
Surgical levels, %				1.000	0.607
Thoracic	14 (43.75%)	7 (43.75%)	7 (43.75%)		
Thoracolumbar	12 (37.50%)	5 (31.25%)	7 (43.75%)		
Lumbar	6 (18.75%)	4 (25.00%)	2 (12.50%)		
Operation time, min	192.97 ± 48.77	188.75 ± 53.65	197.19 ± 44.72	0.483	0.632
Blood loss, ml	427.19 ± 112.31	431.88 ± 120.68	422.50 ± 107.05	−0.232	0.818
Duration of follow-up, months	45.03 ± 8.00	44.50 ± 8.29	45.56 ± 7.94	0.408	0.714

The CRP and ESR values in the iliac bone group decreased significantly from 29.88 ± 10.14 and 39.25 ± 9.43 preoperatively, to 6.81 ± 2.23 and 9.69 + 3.03 at the final follow-up, respectively (*p* < 0.001). The CRP and ESR values in the n-HA/PA66 group decreased significantly from 33.19 ± 10.89 and 46.63 ± 15.65 preoperatively, to 6.56 ± 2.48 and 9.31 ± 3.34 at the final follow-up, respectively (*p* < 0.001). There were no significant differences in the CRP and ESR values between the two groups at the final follow-up. The VAS scores in the iliac bone and n-HA/PA66 group were significantly improved from 6.94 ± 1.12 and 6.88 ± 1.41 preoperatively to 3.81 ± 0.75 and 4.06 ± 0.77 at the 3-month follow-up postoperatively (both *p* < 0.001) (Table 2). The JOA scores in the iliac bone and n-HA/PA66 group were significantly improved at the 3-month follow-up postoperatively, as compared to the baseline values (14.25 ± 3.47 vs. 7.06 ± 2.77 and 15.31 ± 4.58 vs. 6.63 ± 3.50, both *p* < 0.001) (Table 2). Then, improvements of VAS and JOA scores continue long at final follow-up. The VAS scores in the iliac bone and n-HA/PA66 group at final follow-up were 2.06 ± 0.57 and 2.25 ± 0.68, respectively. The JOA scores continued to improve significantly from 14.25 ± 3.47 and 15.31 ± 4.58 at the 3-month postoperatively to 21.25 ± 3.99 and 23.19 ± 3.43 at the final follow-up. There were significant improvements in both groups with respect to VAS and JOA scores; however, there were no significant differences in the VAS and JOA scores at any time point between the two groups (*p* > 0.05).

**TABLE 2:** Comparison of clinical outcomes between the two group.

	Iliac bone group (n = 16)	n-HA/PA66 group (n = 16)	t	*p*-value
CRP (mg/L)				
Preoperative	29.88 ± 10.14	33.19 ± 10.89	0.891	0.380
Final follow-up	6.81 ± 2.23	6.56 ± 2.48	-0.300	0.766
*p*-value	<0.001	<0.001		
ESR (mm/h)				
Preoperative	39.25 ± 9.43	46.63 ± 15.65	1.614	0.117
Final follow-up	9.69 + 3.03	9.31 ± 3.34	-0.333	0.742
*p*-value	<0.001	<0.001		
VAS				
Preoperative	6.94 ± 1.12	6.88 ± 1.41	-0.139	0.891
Postoperative 3m	3.81 ± 0.75	4.06 ± 0.77	0.929	0.360
Final follow-up	2.06 ± 0.57	2.25 ± 0.68	0.841	0.407
*p*-value^a^	<0.001	<0.001		
*p*-value^b^	<0.001	<0.001		
JOA				
Preoperative	7.06 ± 2.77	6.63 ± 3.50	-0.392	0.698
Postoperative 3m	14.25 ± 3.47	15.31 ± 4.58	0.742	0.464
Final follow-up	21.25 ± 3.99	23.19 ± 3.43	1.473	0.151
*p*-value^a^	<0.001	<0.001		
*p*-value^b^	<0.001	<0.001		

^a^ , comparison between preoperative and postoperative 3m

^b^ , comparison between final follow-up and postoperative 3m

**FIGURE 1:**
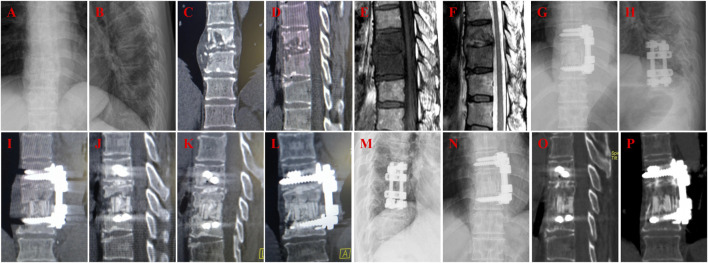
A 57-year-old female with T9-10 tuberculosis in n-HA/PA66 group and received one-stage lesion debridement, n-HA/PA66 composite cage placement, allogeneic bone interbody fusion, and instrumentation. **(A–F)** Preoperative X-ray, 3D CT and MRI showing destruction of T10-11 intervertebral disc and vertebral bodies. **(G-H)** X-ray immediate after surgery. **(I-J)** 3D CT at 3 months after surgery showing good internal fixation position. **(K–L)** 3D CT at 12 months after surgery showing good internal fixation position and solid bone fusion. **(M–P)** 3D CT at 45 months after surgery showing good internal fixation position and solid bone fusion was obtained.

**FIGURE 2:**
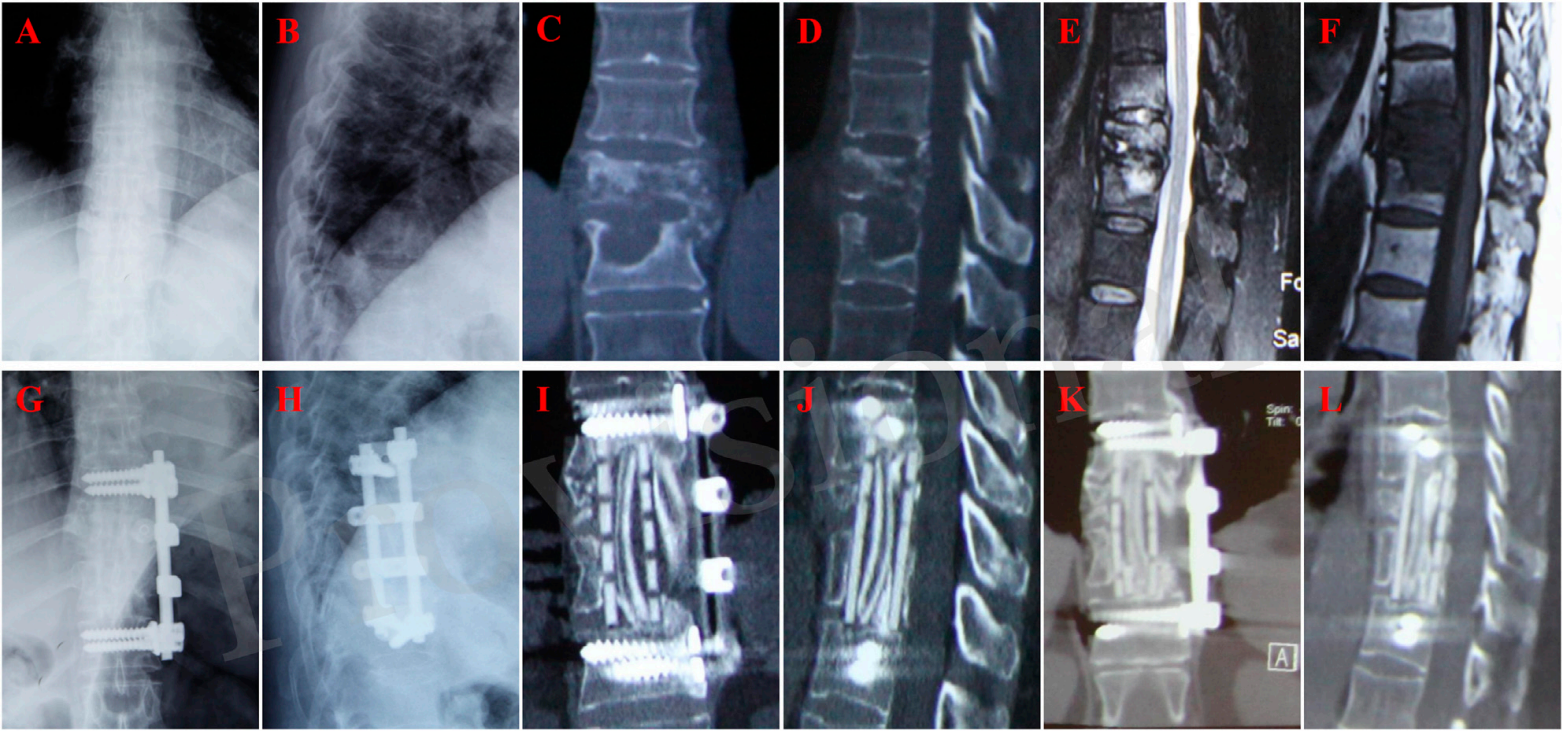
A 60-year-old female with T10-11 tuberculosis in n-HA/PA66 group and received one-stage lesion debridement, n-HA/PA66 composite cage placement, allogeneic bone interbody fusion, and instrumentation. **(A–F)** Preoperative X-ray, 3D CT and MRI showing destruction of T10-11 intervertebral disc and adjacent vertebral bodies. **(G–H)** X-ray immediate after surgery. **(I, J)** 3D CT at 12 months after surgery showing good internal fixation position. **(K, L)** 3D CT at 46 months after surgery showing solid bone fusion and no significant subsidence of the graft.

**FIGURE 3:**
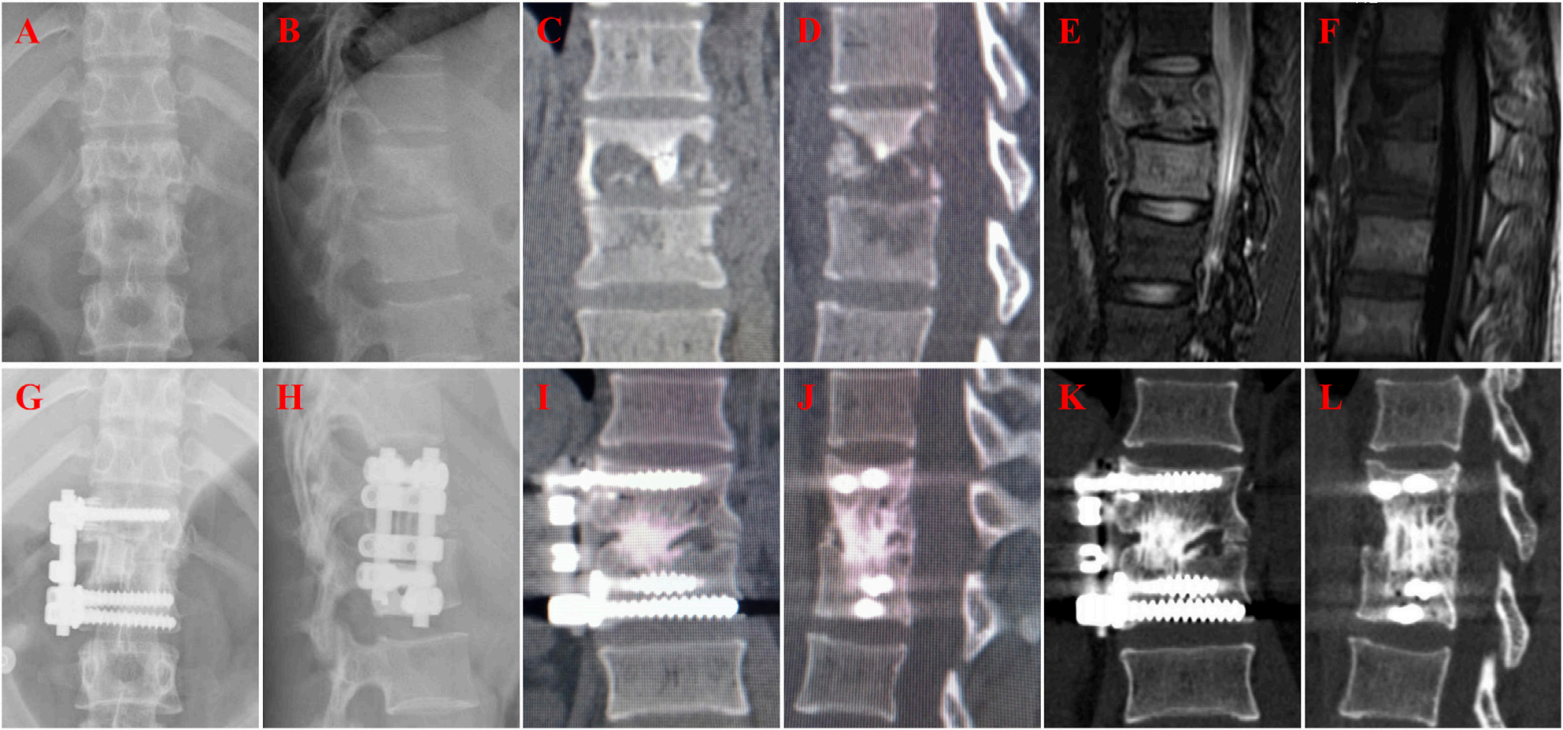
A 22-year-old female with T12-L1 tuberculosis in iliac bone graft group and received one-stage lesion debridement, autogenous iliac bone placement, interbody fusion and instrumentation. **(A–F)** Preoperative X-ray, 3D CT and MRI showing destruction of T12-L1 intervertebral disc and vertebral bodies. **(G, H)** X-ray immediate after surgery. **(I, J)** 3D CT at 37 months after surgery showing good internal fixation position and solid bone fusion. **(K,L)** 3D CT at 58 months after surgery showing solid bone fusion and no significant subsidence of the graft.

**FIGURE 4:**
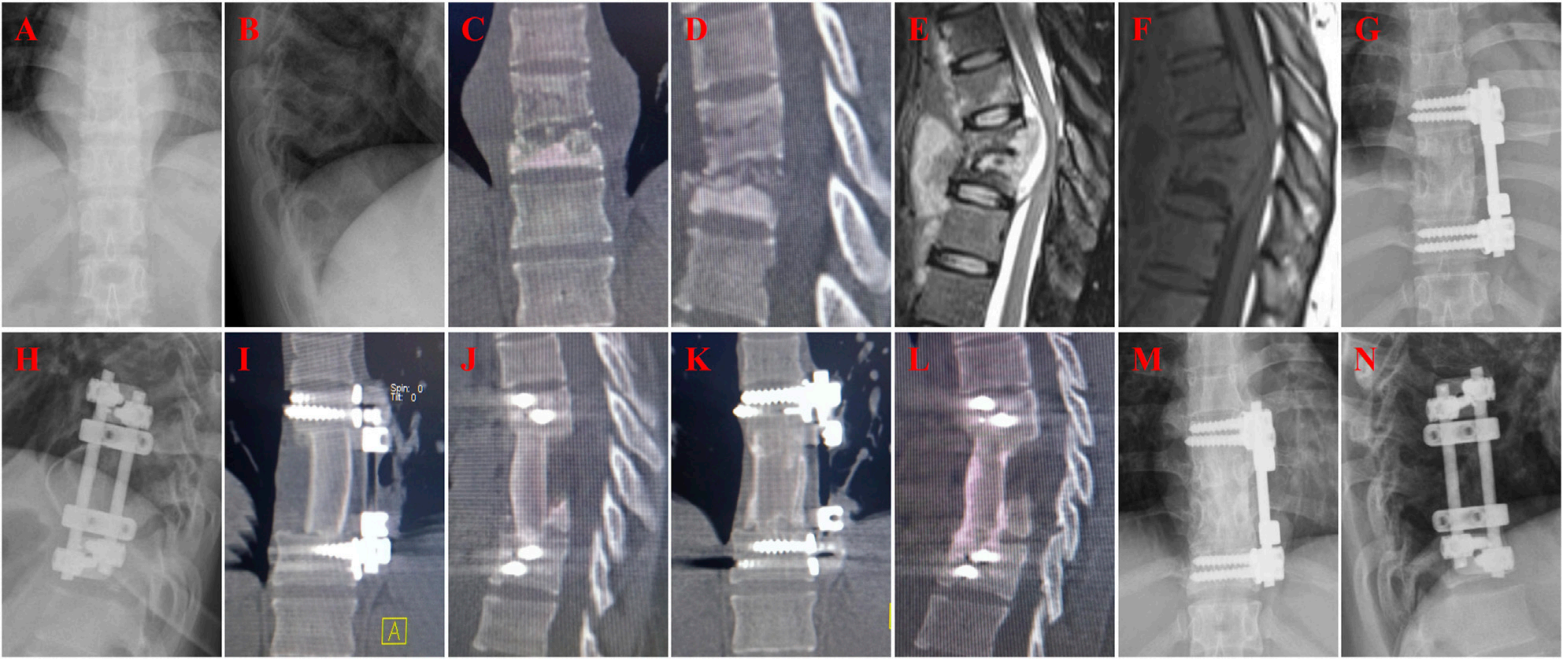
A 54-year-old female with T10-11 tuberculosis in iliac bone graft group and received one-stage lesion debridement, autogenous iliac bone placement, interbody fusion and instrumentation. **(A–F)** Preoperative X-ray, 3D CT and MRI showing destruction of T10-11 intervertebral disc and adjacent vertebral bodies. **(G, H)** X-ray immediate after surgery. **(I, J)** 3D CT at 3 months after surgery showing good internal fixation position. **(K, L)** 3D CT at 12 months after surgery showing good fusion and no significant subsidence of the graft. **(M, N)** X-ray at 61 months after surgery showing good internal fixation position and solid bone fusion.

Although the SA significantly increased after surgery in both groups, there was no significant difference at any time point after surgery (*p* > 0.05) (Table 3). More specifically, the SA at postoperatively 1-week and final follow-up of n-HA/PA66 group is less than that of iliac bone group (16.63 vs. 17.88 postoperatively 1-week, 20.50 vs. 22.38 at final follow-up). Then, the SA was slightly decreased at the final follow-up compared with that at 1 week after surgery. The mean correction of SA was 12.94 ± 4.37 in the iliac bone group, and 12.19 ± 5.88 in the n-HA/PA66 group, and the mean loss of SA was 4.50 ± 1.27 and 3.88 ± 1.03 for each group, respectively (Table 3). There were no significant differences in the cage subsidence and fusion time between the two groups (*p* > 0.05) (Table 3). Figures 1–4 showed the representative cases for the n-HA/PA66 group and the iliac bone group, respectively.

**TABLE 3:** Comparison of radiographic parameters between the two group.

	Iliac bone group (n = 16)	n-HA/PA66 group (n = 16)	t	*p*-value
SA (°)				
Preoperative	30.81 ± 10.41	28.81 ± 9.92	−0.556	0.582
Postoperative 1w	17.88 ± 6.77	16.63 ± 6.15	−0.547	0.589
Final follow-up	22.38 ± 7.15	20.50 ± 6.39	−0.783	0.440
Correction	12.94 ± 4.37	12.19 ± 5.88	−0.409	0.685
Loss	4.50 ± 1.27	3.88 ± 1.03	−1.536	0.135
Cage subsidence (mm)	1.26 ± 1.23	1.52 ± 1.66	0.508	0.615
Fusion time (months)	8.88 ± 2.63	7.75 ± 2.30	−1.289	0.207

**FIGURE 5:**
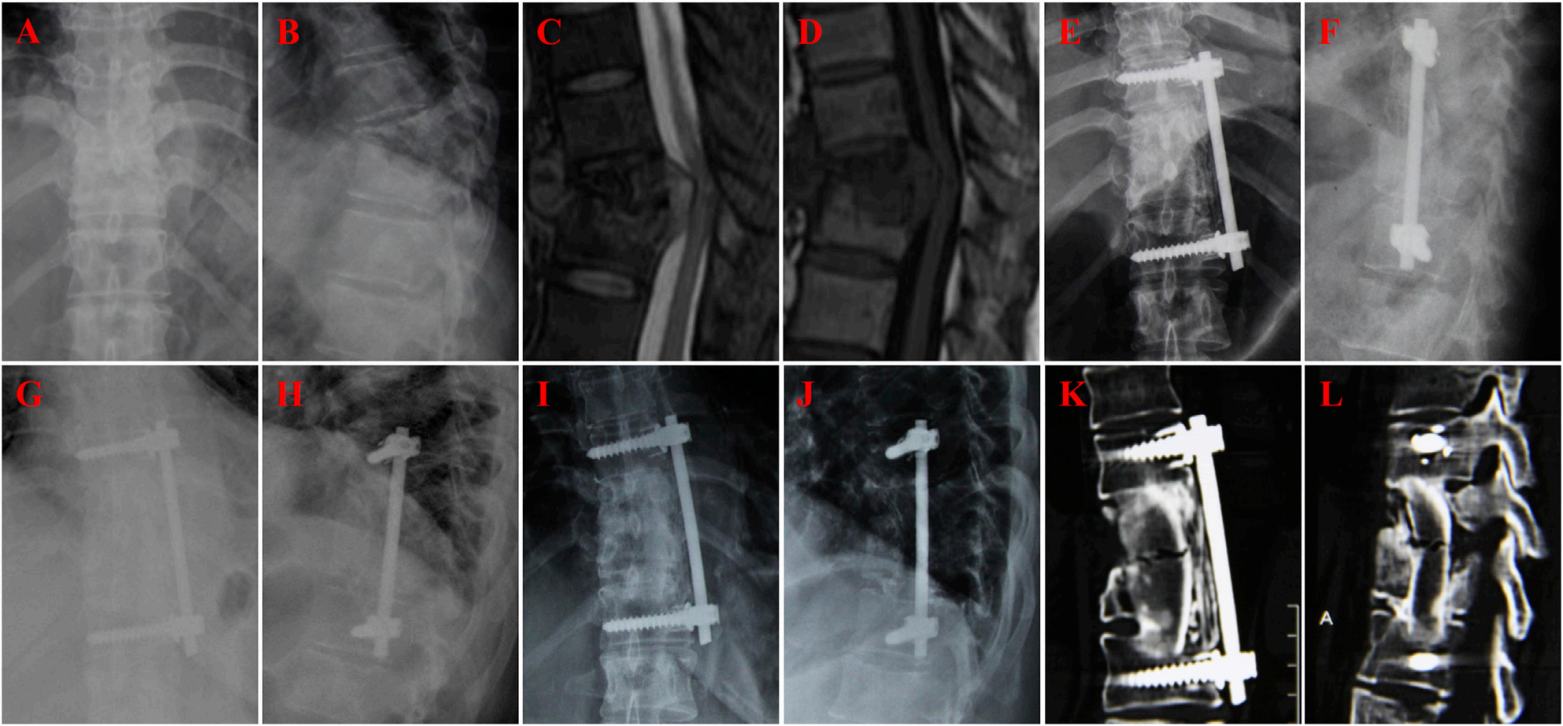
A 63-year-old female with T10-11 tuberculosis in iliac bone graft group and received one-stage lesion debridement, autogenous iliac bone placement, interbody fusion and instrumentation. **(A–D)** Preoperative X-ray and MRI showing destruction of T10-11 intervertebral disc and adjacent vertebral bodies. **(E, F)** X-ray immediate after surgery. **(G, H)** X-ray at 3 months after surgery showing good internal fixation position. **(I, J)** X-ray at 13 months after surgery showing good internal fixation position and no significant subsidence of the graft. **(K, L)** 3D CT at 13 months after surgery showing good fusion at both ends of the iliac bone, but a fracture in the middle of the iliac bone.

In terms of neurological function, there were no patients with ASIA grade A or B in all cases included in this study. In the iliac bone group: 1 case improved from grade B to C; among 3 grade C cases preoperative, 1 case improved from grade C to D and 2 cases improved to grade E; 12 cases improved from grade D to E at the last follow-up. In the n-HA/PA66 group: 1 case improved from grade B to C; among 4 grade C cases preoperative, 1 case maintained grade C, 3 cases improved from grade C to D; 11 cases improved from grade D to E at the last follow-up (Table 4). There was no statistical significance in the improving of neurological function between the two groups. Changes in ASIA classification preoperatively and at the last follow up are shown in Table 4. The total complications were not different between the two groups. There were one case of wound infection, and one case of graft broken in iliac bone group (Figure 5), and one case of wound infection in n-HA/PA66 group (Table 5). All cases resolved completely by conservative treatment.

**TABLE 4:** Comparison of ASIA grading of neurological function between the two group.

Group	Preoperative	N	Final follow-up				
			A	B	C	D	E
Iliac bone group	A	0	0	0	0	0	0
	B	1	0	0	1	0	0
	C	3	0	0	0	1	0
	D	12	0	0	0	0	14
	E	0	0	0	0	0	0
n-HA/PA66 group	A	0	0	0	0	0	0
	B	1	0	0	0	0	0
	C	4	0	0	0	2	0
	D	11	0	0	0	0	14
	E	0	0	0	0	0	0

**TABLE 5:** Complications between the two groups.

Parameters	Iliac bone group (n = 16)	n-HA/PA66 group (n = 16)	t/χ^2^	*p*-value
Perioperatively				
Wound infection	1	1		
Nerve root injury	0	0		
Implant-related				
Screw loosening	0	0		
Screw broken	0	0		
Graft broken	1	0		
Total complications	2	1	0.368	0.544

## Discussion

Autogenous iliac bone graft has been the gold standard of bone graft fusion in clinic because of its high support strength, mild immune rejection and high fusion rate (Fu et al., 2016; Xia et al., 2023). But its bone mass is limited, unable to satisfy the multi-level vertebral body destruction reconstruction. Moreover, tuberculosis patients have poor spinal bone condition due to long-term pain, immobilization, malnutrition and adjacent segment tuberculosis involvement (Pigrau-Serrallach and Rodriguez-Pardo, 2013). The risk of long-term loss of support subsidence correction in spinal tuberculosis is greater. As a bioactive material, n-HA/PA66 has the good biocompatibility of polyamide 66 (PA66) and the good osteogenic conductivity and strong mechanical properties of nano-hydroxyapatite (HA) (similar to those of human cortical bone) (Wang et al., 2007). In recent years, n-HA/PA66 support has been more and more widely used in anterior column reconstruction surgery and achieved satisfactory clinical results (Yang et al., 2014; Hu et al., 2019; Deng et al., 2022; Li et al., 2023; Zhang et al., 2023). In this study, we retrospectively analyzed 16 patients who underwent anterior reconstruction of thoracolumbar tuberculosis using n-HA/PA66 cage and matched them with 16 patients who used ilium bone. With at least 4 years of follow-up, n-HA/PA66 showed some advantages with good mid-term radiographic and clinical results.

In implant-associated infections, bacteria in biofilms are resistant to antibiotics or host defense mechanisms (Martinez-Perez et al., 2017; Xi et al., 2021). It is often necessary to remove the implant to eradicate the infection. In contrast, in tuberculous infections, complete debridement and antituberculous chemotherapy without implant removal is considered a safe approach. In contrast to *Staphylococcus* epidermidis, *M. tuberculosis* adherence to implant surfaces and biofilm formation are less likely, providing the basis for successful internal fixation of spinal tuberculosis (Ha et al., 2005; Chen et al., 2011). MubarakAli et al. (MubarakAli et al., 2023) synthesized HA nanoparticles and found that they effectively inhibit the formation of biofilms by Gram-negative bacteria such as *Pseudomonas aeruginosa*, indicating the potential antibacterial properties of HA materials. Research by Sun et al. (Sun et al., 2023) also demonstrated that a Sim-HA coating of titanium alloy effectively inhibits the formation of biofilms by *Staphylococcus aureus* while enhancing osteogenesis and osseointegration. Therefore, the material has good biocompatibility with human body, can promote the distribution of human immune cells, antibodies and antibacterial drugs in the material, can effectively eliminate tuberculosis bacilli and play an antibacterial role, which provides a biological basis for the application of the material in infectious lesions such as tuberculosis.

The elastic modulus of n-HA/PA66 support is close to human cortical bone, and it has biomechanical properties matching with human bone (Xiong et al., 2014; Zou et al., 2017). It can not only meet the support strength of vertebral body, but also effectively reduce the stress shielding effect in bone reconstruction (Xu et al., 2010). The three-dimensional porous design of n-HA/PA66 biomimetic scaffold ensures a high porosity, which promotes the adhesion, proliferation, and differentiation of bone cells, facilitating the crawl growth of the induced new bone tissue (Yang et al., 2014). After the n-HA/PA66 cage was implanted, it could release calcium and phosphorus ions at the material-tissue interface, and provide an ideal microenvironment for osteogenesis, which may conducive to the osteoconductive growth of bone graft and early fusion (Wang et al., 2002; Xu et al., 2010; Qian et al., 2013). This is the reason that the outcomes of n-HA/PA66 cage group are comparable to those of the iliac bone group, with a similar high fusion rate.

There are many causes of cage subsidence, including osteoporosis, endplate manipulation, cage size, cage position, and the material characteristics of the cage (Niu et al., 2002; Zhang et al., 2018; Pinter et al., 2023). The residual vertebral bodies of tuberculosis patients have already removed the bone endplates, and the bone strength is weak, which may be one of the important reasons for the subsidence of the bone endplates. Widened edge design reduces support cutting of endplates. In addition, the prefabricated serrations at the upper and lower ends of the support can help prevent displacement (Yang et al., 2013). Combined with anterior fixation, it can reduce the occurrence of support displacement and dislocation. In our study, there was no significant difference in cage subsidence between the n-HA/PA66 and iliac bone groups. This could explain the early fusion of the n-HA/PA66 cage.

The mid-term radiographic and clinical results were satisfactory in both the n-HA/PA66 and iliac groups. Although the SA significantly increased after surgery in both groups, there was no significant difference at any time point after surgery. The VAS and JOA scores improved significantly postoperatively and remained good at final follow-up. After fusion, the bone remodeling process is associated with cage access to the vertebral body. The study by Kim et al. (Kim et al., 2012) reported that cage subsidence did not have any effect on clinical outcomes. Similarly, in our series, all patients with cage subsidence did not show worse or poorer clinical outcomes at postoperative follow-up. This also explains the lack of significant difference in postoperative VAS scores between the two groups of patients in our study.

The present study first reported the long-term outcomes of two implant materials in spinal tuberculosis bone graft fusion. The n-HA/PA66 cage could achieve similar outcomes as autogenous iliac bone for anterior reconstruction application of spinal defect for thoracolumbar tuberculosis. However, there are still some limitations of our study. This is a retrospective study in a single center, and small sample was another limitation for the study. Further prospective large scale randomized controlled trials are needed to confirm the current findings.

## Conclusion

This retrospective study demonstrated satisfactory long-term clinical results with n-HA/PA66 cage in the treatment of spinal tuberculosis bone graft fusion were obtained, and a similar high fusion rate as autogenous iliac bone. n-HA/PA66 cages are an ideal material comparable to autogenous iliac bone.

## Data Availability

The raw data supporting the conclusion of this article will be made available by the authors, without undue reservation.
